# Correction to: Expression of the SARS‑CoV‑2 receptorACE2 in human heart is associated with uncontrolled diabetes, obesity, and activation of the renin angiotensin system

**DOI:** 10.1186/s12933-021-01402-7

**Published:** 2021-10-28

**Authors:** Michal Herman-Edelstein, Tali Guetta, Amir Barnea, Maayan Waldman, Naomi Ben-Dor, Yaron D. Barac, Ran Kornowski, Michael Arad, Edith Hochhauser, Dan Aravot

**Affiliations:** 1 Cardiac Research Laboratory, Felsenstein Medical Research Center, Sackler School of Medicine Tel-Aviv University, Tel Aviv, Israel; 2 Department of Cardiothoracic Surgery, Rabin Medical Center, Petach Tikva, Israel; 3 Department of Cardiology, Rabin Medical Center, 49100 Petach Tikva, Israel; 4 Leviev Heart Center, Sheba Medical Center, Tel Hashomer, Tel Aviv University, Tel Aviv, Israel; 5 Nephrology Department, Rabin Medical Center, Petach Tikva, Israel; 6 Sackler Faculty of Medicine, Tel Aviv University, Tel Aviv, Israel

## Correction to: Cardiovasc Diabetol (2021) 20:90 10.1186/s12933-021-01275-w

Following publication of the original article [1], the author name “Yaron D. Barac” was incorrectly written as “Yaron Barak”. This has been corrected with this erratum.

The original article has also been corrected.
